# Crystal structure of *N*,*N*,*N*′,*N*′-tetra­methyl­ethane­di­amine

**DOI:** 10.1107/S2056989021012457

**Published:** 2022-01-01

**Authors:** Tobias Schrimpf, Felix Otte, Carsten Strohmann

**Affiliations:** a Technische Universität Dortmund, Fakultät Chemie und Chemische Biologie, Otto-Hahn-Strasse 6, 44227 Dortmund, Germany

**Keywords:** crystal structure, Hirshfeld surface analysis, bidentate ligand

## Abstract

The title compound *N*,*N*,*N*′,*N*′-tetra­methyl­ethanedi­amine, C_6_H_16_N_2_, is a bidentate amine ligand commonly used in organolithium chemistry for deaggregation. Crystals were grown at 243 K from *n*-pentane solution. The complete mol­ecule is generated by a crystallographic center of symmetry and the conformation of the di­amine is anti­periplanar. To investigate the inter­molecular inter­actions, a Hirshfeld surface analysis was performed. It showed that H⋯H (van der Waals) inter­actions dominate with a contact percentage of 92.3%.

## Abstract 2

*N*,*N*,*N*′,*N*′-tetra­methyl­ethanedi­amine, C_6_H_16_N_2_, crystallizes in the monoclinic crystal system in the space group *P*2_1_/c. For the investigation of the conformation, quantum chemical methods were used and for inter­molecular inter­actions, a Hirshfeld surface analysis was performed.

## Chemical context

*N*,*N*,*N*′,*N*′-tetra­methyl­ethanedi­amine (TMEDA, C_6_H_16_N_2_, **1**) consists of two tertiary amine groups linked by an ethyl­ene bridge. It can be used in cross-coupling or in olefin polymerization reactions where, *e.g.*, a complex between di­methyl­nickel and TMEDA is used as a catalyst (Göttker-Schnetmann & Mecking, 2020 ▸). However, TMEDA is most commonly used in the chemistry of organolithium compounds. The lithium–carbon bond is characterized by its high polarity, as it contains a cationic lithium and carbanionic residues. These organolithium compounds form unreactive aggregates in non-polar solvents, which can be deaggregated by adding Lewis-basic ligands (Gessner *et al.*, 2009 ▸). Compound **1** can be used as such a ligand, which can either chelate the metal center to form commonly dimeric structures or bridge two or more metal centers to form coordination polymers. The dimeric structural motif can be obtained in the butyl lithium TMEDA complex (Nichols & Williard, 1993 ▸), the enolate structure (Nichols *et al.*, 2007 ▸) and in the phen­yl(ethyn­yl) lithium (Schubert & Weiss, 1983 ▸), whereas the lithium diiso­propyl­amide forms a polymeric structure with **1** bridging the lithium amide groups (Bernstein *et al.*, 1992 ▸) (see scheme). The main benefit of deaggregation is the increased reactivity of organolithium compounds. Accompanying with smaller aggregates, the carbanionic center is more accessible for substrates due to an available coordination site at the metal center (Gessner *et al.*, 2009 ▸). In the case of sterically more demanding ligands, however, the reactivity can even be reduced, since the coordination site at the lithium center can be sterically shielded (Knauer *et al.*, 2019 ▸). Quantum chemical considerations of aggregation and deaggregation require knowledge of the energetically most favorable conformer. With knowledge about this conformer, quantum chemical equilibria can be used to calculate reasonable energies, for example to predict the reactivity or the formation of certain aggregates. 
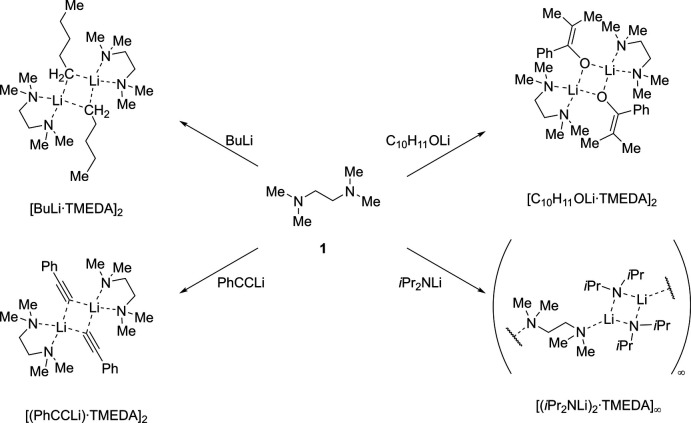


## Structural commentary

**Figure 1:**
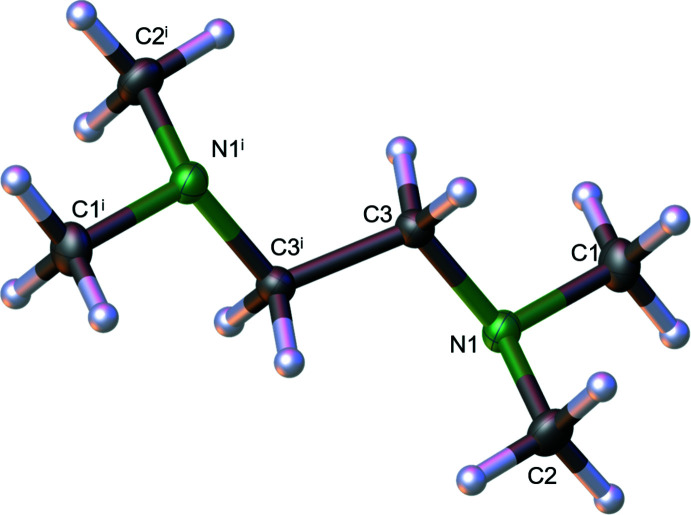
Crystal structure of **1** showing 50% displacement ellipsoids. Symmetry code: (i) −*x*, 1 − *y*, 1 − *z*.

Compound **1** crystallizes from *n-*pentane solution at 243 K in the monoclinic crystal system in space group *P*2_1_/*c*. The asymmetric unit consists of half of the mol­ecule, with the other half generated by crystallographic inversion symmetry. The mol­ecular structure of **1** is presented in Fig. 1 ▸ and selected bond angles are given in Table 1 ▸. The bond lengths for **1** are typical for C—C and C—N bonds and show no irregularities. The ethyl­ene fragment is arranged in a staggered conformation where the nitro­gen atoms are arranged in an anti­periplanar arrangement with N1—C3—C3^i^—N1^i^ [symmetry code: (i) −*x*, 1 − *y*, 1 − *z*] = 180.0° by symmetry. The conformations of the C1—N1—C3—C3^i^ and C2—N1—C3—C3^i^ groupings are *anti* [torsion angle = 167.33 (6)°] and *gauche* [–71.17 (8)°], respectively.

**Figure 2:**
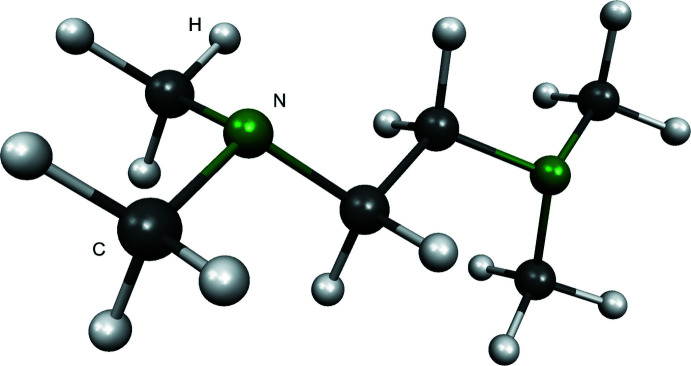
Structure of **1** obtained by geometry optimization at the M062X/6–31+g(d,p) theory level.

A quantum chemical geometry optimization was performed at the M062X/6-31+G(d,p) (Walker *et al.*, 2013 ▸) theory level using *Gaussian 16* (Frisch *et al.*, 2016 ▸). The calculated geometry is shown in Fig. 2 ▸ and the bond angles are shown in Table 1 ▸. The angles of C2—N1—C1 and C2—N1—C3 differ by about 1° between the crystal structure and the quantum chemical calculated structure. Since these are gas phase calculations, this difference can be neglected. However, the N1—C3—C3 bond angles are in the same range. Therefore, we may assume that the presented conformation is at a local energy minimum.

## Supra­molecular features

**Figure 3:**
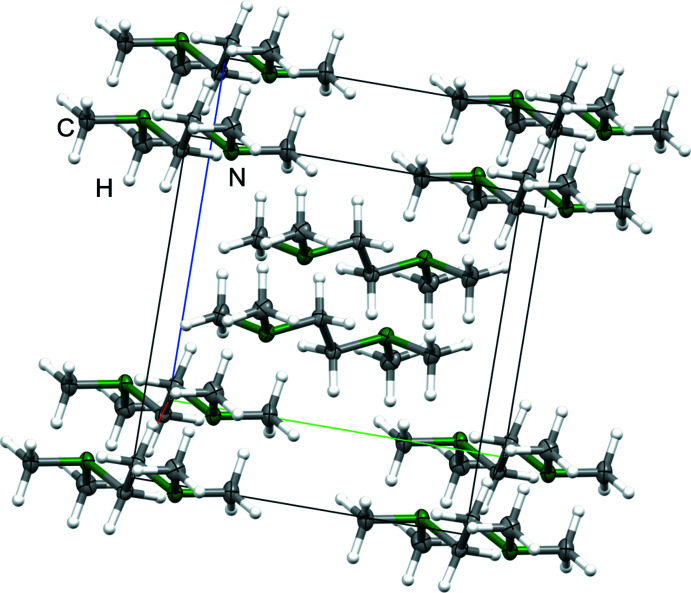
A view along the *b*-axis direction of the crystal packing of **1**.

**Figure 4:**
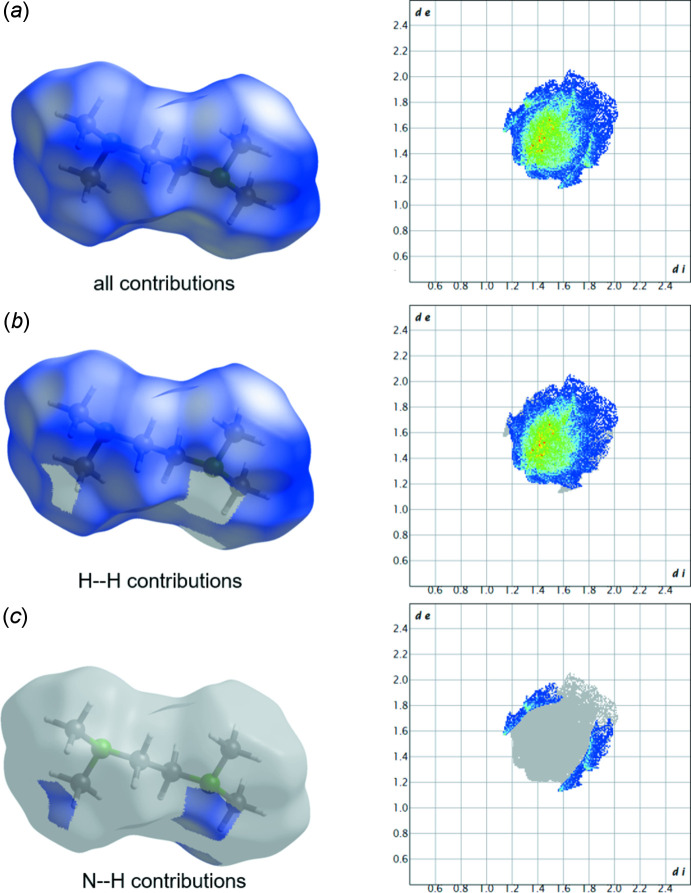
Two-dimensional fingerprint plots of **1**, (*a*) showing all contributions, (*b*) showing the H/H contributions and (*c*) showing the contributions of nitro­gen and hydrogen (blue areas). The corresponding surfaces obtained by Hirshfeld surface analysis are also displayed.

The crystal packing of compound **1** is shown in Fig. 3 ▸. For the investigation of close contacts and inter­molecular inter­actions, a Hirshfeld surface analysis (Spackman & Jayatilaka, 2009 ▸) was carried out: Fig. 4 ▸ illustrates the Hirshfeld surface mapped over *d*_norm_ in the range from −0.072 to 1.201 arbitrary units and the related fingerprint plots generated by *CrystalExplorer* (Spackman *et al.*, 2021 ▸; McKinnon *et al.*, 2007 ▸). Weak van der Waals H⋯H contacts are the largest region (92.3%). The remaining 6.7% are generated by N⋯H contacts, whereby C⋯H contacts do not contribute to crystal packing. In addition, no red spots are visible, which leads to the conclusion that the packing of the crystal is caused only by van der Waals inter­actions. The absence of packing effects such as hydrogen bonds also suggests that the most energetically favorable conformer is present.

## Database survey

There are a large number of compounds where **1** is used as a ligand. Selected examples found in the Cambridge Structural Database (CSD, version 5.41, update of May 2020; Groom *et al.*, 2016 ▸) include dilithium bis­(tri­methyl­sil­yl)-*o*-xylene bis(tetra­methyl­ethanedi­amine) (CSD refcode BECWEL; Lappert *et al.*, 1982 ▸), allyl lithium *N*,*N*,*N*′,*N*′-tetra­methyl­ethanedi­amine (BITNEX; Köster & Weiss, 1982 ▸), tetra­sodium tetra­kis­(tetra­methyl­ethylenedi­amine) octa­chloro­ditungsten (BORZUD; Cotton *et al.*, 1982 ▸), sodium (2,2,6,6-tetra­methyl­piperidin-1-ide)ferrocenyl-*t*-butyl-zinc *N*,*N*,*N*′,*N*′-tetra­methyl­ethane-1,2-di­amine solvate (BUQJII; Clegg *et al.*, 2015 ▸), hexa­kis­(*μ_2_*-meth­yl)-tris­(tetra­methyl­ethylenedi­amine­lithium)methyl­thorium(IV) tetra­methyl­ethylenedi­amine (COSZOZ; Lauke *et al.*, 1984 ▸), dilithium tri­benzyl­idene­methane bis­(tetra­methyl­ethylenedi­amine) (COZJUW; Wilhelm *et al.*, 1984 ▸), cyclo­penta­dienyl sodium tetra­methyl­ethylenedi­amine (CPNATM10; Aoyagi *et al.*, 1979 ▸).

Since **1** plays a major role in organolithium chemistry, it also finds application in the group of Strohmann *et al.* Thus, some publications are included here: *t*-butyl lithium *N*,*N*,*N*′,*N*′-tetra­methyl­ethanedi­amine (Gessner & Strohmann, 2008 ▸), isopropyl lithium *N*,*N*,*N*′,*N*′-tetra­methyl­ethanedi­amine (Stroh­mann *et al.*, 2008 ▸), (di­ethyl­amino)­diphenyl­sil­yl) *N*,*N*,*N*′,*N*′-tetra­methyl­ethanedi­amine (Strohmann *et al.*, 2006 ▸), [(*R*)-({[(*S*)-2-(meth­oxy­meth­yl)pyrrolidin-1-yl]meth­yl}di­meth­yl­sil­yl)(phen­yl)meth­yl]lithium *N*,*N*,*N*′,*N*′-tetra­methyl­ethane­di­amine (Strohmann *et al.*, 2003 ▸), methyl lithium *N*,*N*,*N*′,*N*′-tetra­methyl­ethanedi­amine (Gessner *et al.*, 2011 ▸) and zinc bromide *N*,*N*,*N*′,*N*′-tetra­methyl­ethanedi­amine (Eckert *et al.*, 2013 ▸).

## Synthesis and crystallization

*N*,*N*,*N*′,*N*′-Tetra­methyl­ethanedi­amine (C_6_H_16_N_2_, **1**) was purchased by Sigma-Aldrich and was used without further purification. A solution of TMEDA (0.5 mmol) in *n*-pentane (1 ml) was prepared at 243 K and **1** crystallized in the form of colorless blocks.

## Refinement

Crystal data, data collection and structure refinement details are summarized in Table 2 ▸. For both compounds, the H atoms were positioned geometrically (C—H = 0.95–1.00 Å) and refined using a riding model, with *U*_iso_(H) = 1.2*U*_eq_(C) for CH_2_ and CH hydrogen atoms and *U*_iso_(H) = 1.5*U*_eq_(C) for CH_3_ hydrogen atoms.

## Supplementary Material

Crystal structure: contains datablock(s) I. DOI: 10.1107/S2056989021012457/hb8002sup1.cif

Structure factors: contains datablock(s) I. DOI: 10.1107/S2056989021012457/hb8002Isup2.hkl

Supporting information file. DOI: 10.1107/S2056989021012457/hb8002Isup3.cml

CCDC reference: 2123810

Additional supporting information:  crystallographic information; 3D view; checkCIF report

## Figures and Tables

**Table 1:** Comparison of the bond angles (°) between the crystal structure and the quantum computed structure.

Atoms	Crystal structure	Calculated
C2—N1—C1	109.26 (5)	110.29
C2—N1—C3	111.96 (5)	110.26
N1—C3—C3′	112.37 (6)	112.32

**Table 2:** Experimental details

Crystal data	
Chemical formula	C_6_H_16_N_2_
*M*_r_	116.21
Crystal system, space group	Monoclinic, *P*2_1_/*c*
Temperature (K)	100
*a*, *b*, *c* (Å)	5.6987 (13), 8.311 (2), 8.453 (2)
β (°)	106.954 (9)
*V* (Å^3^)	382.92 (18)
*Z*	2
Radiation type	Mo *K*α
μ (mm^−1^)	0.06
Crystal size (mm)	0.56 × 0.35 × 0.30

Data collection	
Diffractometer	Bruker Venture D8
Absorption correction	Multi-scan (*SADABS*; Krause *et al.*, 2015 ▸)
*T*_min_, *T*_max_	0.371, 0.567
No. of measured, independent and observed [*I* > 2σ(*I*)] reflections	16732, 1720, 1434
*R*_int_	0.037
(sin θ/λ)_max_ (Å^−1^)	0.813

Refinement	
*R*[*F* ^2^ > 2σ(*F* ^2^)], *wR*(*F* ^2^), *S*	0.038, 0.110, 1.05
No. of reflections	1720
No. of parameters	39
H-atom treatment	H-atom parameters constrained
Δρ_max_, Δρ_min_ (e Å^−3^)	0.41, −0.15

Computer programs: *APEX2* and *SAINT* (Bruker, 2018 ▸), *SHELXT* (Sheldrick, 2015*a* ▸), *SHELXL2014/7* (Sheldrick, 2015*b* ▸) and *OLEX2* (Dolomanov *et al.*, 2009 ▸).

## supplementary crystallographic information

### Crystal data

**Table:**

C_6_H_16_N_2_	*F*(000) = 132
*M**_r_* = 116.21	*D*_x_ = 1.008 Mg m^−^^3^
Monoclinic, *P*2_1_/*c*	Mo *K*α radiation, λ = 0.71073 Å
*a* = 5.6987 (13) Å	Cell parameters from 352 reflections
*b* = 8.311 (2) Å	θ = 3.5–22.8°
*c* = 8.453 (2) Å	µ = 0.06 mm^−^^1^
β = 106.954 (9)°	*T* = 100 K
*V* = 382.92 (18) Å^3^	Block, colourless
*Z* = 2	0.56 × 0.35 × 0.30 mm

### Data collection

**Table:**

Bruker Venture D8 diffractometer	1720 independent reflections
Radiation source: microfocus sealed X-ray tube, Incoatec Iµs	1434 reflections with *I* > 2σ(*I*)
HELIOS mirror optics monochromator	*R*_int_ = 0.037
Detector resolution: 10.4167 pixels mm^-1^	θ_max_ = 35.3°, θ_min_ = 3.5°
φ and ω scans	*h* = −9→9
Absorption correction: multi-scan (SADABS; Krause *et al.*, 2015)	*k* = −13→13
*T*_min_ = 0.371, *T*_max_ = 0.567	*l* = −12→13
16732 measured reflections	

### Refinement

**Table:**

Refinement on *F*^2^	Primary atom site location: dual
Least-squares matrix: full	Hydrogen site location: inferred from neighbouring sites
*R*[*F*^2^ > 2σ(*F*^2^)] = 0.038	H-atom parameters constrained
*wR*(*F*^2^) = 0.110	*w* = 1/[σ^2^(*F*_o_^2^) + (0.0452*P*)^2^ + 0.073*P*] where *P* = (*F*_o_^2^ + 2*F*_c_^2^)/3
*S* = 1.05	(Δ/σ)_max_ < 0.001
1720 reflections	Δρ_max_ = 0.41 e Å^−^^3^
39 parameters	Δρ_min_ = −0.15 e Å^−^^3^
0 restraints	

### Special details

**Table:**

Geometry. All esds (except the esd in the dihedral angle between two l.s. planes) are estimated using the full covariance matrix. The cell esds are taken into account individually in the estimation of esds in distances, angles and torsion angles; correlations between esds in cell parameters are only used when they are defined by crystal symmetry. An approximate (isotropic) treatment of cell esds is used for estimating esds involving l.s. planes.

### Fractional atomic coordinates and isotropic or equivalent isotropic displacement parameters (Å^2^)

**Table:**

	*x*	*y*	*z*	*U*_iso_*/*U*_eq_	
N1	0.25269 (10)	0.34528 (6)	0.53636 (6)	0.01749 (12)	
C1	0.49338 (12)	0.33807 (9)	0.65977 (9)	0.02394 (15)	
H1A	0.5794	0.4404	0.6608	0.036*	
H1B	0.5896	0.2505	0.6320	0.036*	
H1C	0.4725	0.3185	0.7692	0.036*	
C2	0.12526 (13)	0.19303 (8)	0.53563 (9)	0.02363 (14)	
H2A	0.0976	0.1764	0.6436	0.035*	
H2B	0.2253	0.1048	0.5134	0.035*	
H2C	−0.0328	0.1955	0.4494	0.035*	
C3	0.11367 (10)	0.48144 (7)	0.57161 (7)	0.01782 (13)	
H3A	0.2207	0.5776	0.5952	0.021*	
H3B	0.0631	0.4573	0.6716	0.021*	

### Atomic displacement parameters (Å^2^)

**Table:**

	*U*^11^	*U*^22^	*U*^33^	*U*^12^	*U*^13^	*U*^23^
N1	0.0167 (2)	0.0154 (2)	0.0197 (2)	0.00035 (16)	0.00420 (16)	0.00150 (16)
C1	0.0170 (3)	0.0261 (3)	0.0265 (3)	0.0025 (2)	0.0028 (2)	0.0043 (2)
C2	0.0244 (3)	0.0157 (3)	0.0297 (3)	−0.0014 (2)	0.0063 (2)	0.0015 (2)
C3	0.0176 (2)	0.0163 (2)	0.0174 (2)	0.00014 (19)	0.00177 (17)	−0.00102 (18)

### Geometric parameters (Å, º)

**Table:**

N1—C1	1.4624 (9)	C2—H2A	0.9800
N1—C2	1.4580 (9)	C2—H2B	0.9800
N1—C3	1.4610 (8)	C2—H2C	0.9800
C1—H1A	0.9800	C3—C3^i^	1.5246 (12)
C1—H1B	0.9800	C3—H3A	0.9900
C1—H1C	0.9800	C3—H3B	0.9900
			
C2—N1—C1	109.26 (5)	N1—C2—H2C	109.5
C2—N1—C3	111.96 (5)	H2A—C2—H2B	109.5
C3—N1—C1	109.75 (5)	H2A—C2—H2C	109.5
N1—C1—H1A	109.5	H2B—C2—H2C	109.5
N1—C1—H1B	109.5	N1—C3—C3^i^	112.37 (6)
N1—C1—H1C	109.5	N1—C3—H3A	109.1
H1A—C1—H1B	109.5	N1—C3—H3B	109.1
H1A—C1—H1C	109.5	C3^i^—C3—H3A	109.1
H1B—C1—H1C	109.5	C3^i^—C3—H3B	109.1
N1—C2—H2A	109.5	H3A—C3—H3B	107.9
N1—C2—H2B	109.5		
			
C1—N1—C3—C3^i^	167.33 (6)	N1—C3—C3^i^—N1^i^	180.0
C2—N1—C3—C3^i^	−71.17 (8)		

Symmetry code: (i) −*x*, −*y*+1, −*z*+1.
